# Ultrasonographic evaluation of urinary tract morbidity in school-aged and preschool-aged children infected with *Schistosoma haematobium* and its evolution after praziquantel treatment: A randomized controlled trial

**DOI:** 10.1371/journal.pntd.0005400

**Published:** 2017-02-21

**Authors:** Beatrice Barda, Jean T. Coulibaly, Christoph Hatz, Jennifer Keiser

**Affiliations:** 1 Department of Medical Parasitology and Infection Biology, Swiss Tropical and Public Health Institute, Basel, Switzerland; 2 University of Basel, CH-4003 Basel, Switzerland; 3 Unité de Formation et de Recherche Biosciences, Université Felix Houphouët-Boigny, Abidjan, Côte d’Ivoire; 4 Centre Suisse de Recherches Scientifiques en Côte d’Ivoire, Abidjan, Côte d’Ivoire; 5 Medical Department, Swiss Tropical and Public Health Institute, Basel, Switzerland; Centers for Disease Control and Prevention, UNITED STATES

## Abstract

**Background:**

*Schistosoma haematobium* infections are responsible for significant urinary tract (UT) complications. Schistosomiasis control programs aim to reduce morbidity, yet the extent of morbidity in preschool-aged children and the impact of treatment on morbidity reduction are not well studied.

**Methodology:**

Our study was embedded in a randomized, placebo-controlled, single-blind trial in Côte d’Ivoire, which evaluated the efficacy and safety of three doses (20, 40 and 60 mg/kg) of praziquantel in school-aged (SAC) and preschool-aged (PSAC) children infected with *S*. *haematobium*. Enrolled children were invited to participate in an ultrasound examination prior and six months after treatment. At these time points 3 urine samples were collected for parasitological and clinical examinations.

**Principal findings:**

162 PSAC and 141 SAC participated in the ultrasound examination at baseline, of which 128 PSAC and 122 SAC were present at follow-up. At baseline 43% (70/162) of PSAC had UT morbidity, mostly at bladder level and 7% had hydronephrosis. 67% (94/141) of SAC revealed mainly moderate UT pathology, 4% presented pseudopolyps on the bladder wall, and 6% had pyelectasis. At follow up, 45% of PSAC and 58% of SAC were *S*. *haematobium* positive, mostly harboring light infection intensities (41% and 51%, respectively). Microhematuria was present in 33% of PSAC and 42% of SAC and leukocyturia in 53% and 40% of PSAC and SAC, respectively. 50% (64/128) of PSAC and 58% (71/122) of SAC presented urinary tract morbidity, which was mainly mild. A significant correlation (p<0.05) was observed between praziquantel treatment and reversal of *S*. *haematobium* induced morbidity. Progression of UT pathology decreased with increasing praziquantel dosages. A worsening of morbidity was observed among children in the placebo group.

**Conclusion/Significance:**

Bladder morbidity is widespread among PSAC. Praziquantel treatment is significantly associated with the reversal of *S*. *haematobium* induced morbidity, which underscores the importance of preventive chemotherapy programs. These programs should be expanded to PSAC to prevent or decrease the prevalence of morbidity in young children. This trial is registered as an International Standard Randomized Controlled Trial, number ISRCTN15280205.

## Author summary

*Schistosoma haematobium* is a parasite that infects the human genito-urinary tract. People get infected with the parasite through contact with fresh water and children are at major risk. The complications linked to this infection are due to an inflammation caused by accumulation of the eggs in peri-bladder veins. If not treated, infections can last years and different degrees of severity are observed. These range from thickening of the bladder wall and blurriness of the mucosa to more serious lesions such as pseudo polyps and masses in the bladder that can, with time, evolve in cancer of the bladder. We analyzed preschool-aged children (PSAC) and school-aged children (SAC) with ultrasound before and after praziquantel treatment. Children were randomly assigned to different doses of praziquantel (20, 40 or 60 mg/kg) or to placebo at baseline. Six months after treatment all children underwent another ultrasound of the urinary tract. We included 162 PSAC and 141 SAC at baseline, of which 128 PSAC and 122 SAC had a second ultrasound evaluation six months afterwards. In addition, urine was sampled at both time points for presence of blood, proteins and signs of infection (leukocytes and nitrates). Six months post-treatment 45% of PSAC and 58% of SAC were *S*. *haematobium* positive. Already at the first screening 43% of PSAC and 67% of SAC had bladder lesions. After treatment 50% of PSAC and 58% of SAC still had pathology linked to the infection. We found a correlation between the treatment dose and healing of bladder lesions. On the other hand, we experienced an aggravation of lesions in the placebo group. Praziquantel is given to SAC as preventive chemotherapy every year at national level, where this parasite is endemic. This program should be expanded and include PSAC as well in order to reduce the consequences of infection.

## Introduction

Schistosomiasis primarily caused by *Schistosoma haematobium*, *S*. *japonicum and S*. *mansoni* is a significant public health problem in low-income tropical and subtropical countries. It is an ancient disease with first reports on schistosomiasis dating back 4000 years ago [1]. Yet, still today an estimated 230 million people are infected [2]. Adult *S*. *haematobium* settle in the venous plexus of the genito-urinary tract of the infected host and produce fertilized eggs. Evidence suggests that morbidity is caused by the trapping of eggs within the urinary and genital tract, which induce a granulomatous host immune response. The granuloma formation induces a chronic inflammation resulting in disease manifestations. In more detail, morbidity includes a wide range of pathological presentations, from thickening of the bladder wall mucosa, ureteral dilatation and hydronephrosis, to presence of polyps and masses in the lumen, which could lead to bladder carcinoma in more severe cases [3,4].

*S*. *haematobium* infection is commonly detected by microscopic examination for eggs via urine filtration. Macro and microhematuria and proteinuria are indirect signs of infection, especially in school -aged (SAC) and preschool -aged (PSAC) children [5,6]. In addition, ultrasound examination of the urinary tract (UT) of infected subjects is an important tool to provide information on bladder and kidney lesions [7]. While intensity of infection as well as hematuria are important indirect indicators of morbidity [8,9], UT lesions could be quite different even at similar intensity of infection. Moreover this technique is useful not only at individual level, but also at community level, since it is well accepted, non-invasive and simple to perform [10,11]. Therefore, ultrasonography has been widely used to evaluate morbidity of UT due to *S*. *haematobium* infection [12–15] as well as its resolution after treatment [7,16–18]. It has been shown that UT lesions improve 12 months after treatment and, if not re-treated in case of reinfection, reappear 18 months after treatment [16]. It might be worth highlighting that studies in PSAC have been rare. To date, only few studies have included young children [4,15], which in general show a higher prevalence of morbidities in older children and adolescents. However, given that efforts are ongoing to include PSAC in preventive chemotherapy programs, it is crucial to have more data on the morbidity of PSAC and the impact of praziquantel in the prevention and reversal of morbidity at different follow up times, with the ultimate goal to define suitable control strategies. In addition, the optimal praziquantel dose in PSAC remains to be elucidated and findings on the reversal of morbidity might aid in the selection of optimal treatment dosages.

The aim of our study was therefore to evaluate morbidity in PSAC and SAC infected with *S*. *haematobium* and its resolution 6 months after treatment with different doses of praziquantel compared to placebo.

## Methods

### Ethics statement

Ethical approval for the study was obtained by the National Ethics Committee of the Ministry of Health in Côte d’Ivoire (CNER, reference no. 037/MSLS/CNER-dkn) and the Ethical Committee of Northwestern and Central Switzerland (EKNZ; reference no. 162/2014). Parents/ guardians of enrolled children were informed about the trial, and written informed consent as well as signed assent was obtained before the first child was enrolled. This trial is registered as an International Standard Randomised Controlled Trial, number ISRCTN15280205. All children were treated with praziquantel at the end of the trial according to local guidelines (40 mg/kg).

### Study design and population

Our study was embedded in a randomized, parallel-group, single-blind, placebo-controlled, dose ranging trial in PSAC (aged 2–5 years) and SAC (6–15 years) infected with *S*. *haematobium*. In both cohorts, 40 children per arm were randomized, using block randomization to 20, 40, 60 mg/kg praziquantel or placebo. The ultrasound evaluation was carried out in November 2015 and May 2016, in four different villages (Mopé, Diasson, Nyan, Massandji and Djiougbosso) in the Adzopè region of Côte d’Ivoire.

### Study procedures

Details on the study procedures will be presented elsewhere. Briefly, all children provided three samples of urine on three different days at baseline, 21 days after treatment (follow up; not reported here) and six months after treatment. Urines were examined with the filtration method for detection of *S*. *haematobium* eggs according to standard procedures [19]. In addition, chemical examination of urines was performed using Multistix 10 SG Reagent Strips (Siemens Healthcare, Zurich Switzerland). From each child one stool sample was collected at baseline and 21 days post-treatment for the evaluation of co-infections with *S*. *mansoni* and soil-transmitted helminths. On the day of treatment all children provided one drop of blood for *Plasmodium spp* detection with rapid test (RDT) and hemoglobin measurement.

Before treatment all children underwent a physical examination performed by a physician and body temperature, blood pressure and pulse height and weight were recorded. Signs and symptoms of malaise were assessed with a questionnaire. *S*. *haematobium* egg-positive children fulfilling all inclusion criteria were assigned to one of the four following treatment arms: praziquantel 20 mg/kg (group 1), 40 mg/kg (group 2), 60 mg/kg (group 3) or placebo (group 4). Ultrasound was performed by a trained physician with Sonosite 180 Plus, probe Convex 3.5 mHz ultrasonography machine on the day of treatment. Children were asked to drink at least two full glasses of water before undergoing UT ultrasound. Ultrasound was performed if the bladder was at least 100 cc full and the ureter was considered dilated if its diameter measured >7 mm.

21 days and 6 months post-treatment all treated children were asked to provide three urine samples for detection of *S*. *haematobium* eggs and chemical examination. At the second follow up another sonography of urinary tract was performed from the same operator as at baseline.

### Statistical analysis

Results were double entered in a database (Excel 2010), cross-checked and analyzed with Stata 12.0 (Lakeway Drive College station, TX, Unites States of America). The intensity of infection for *S*. *haematobium* was assessed by calculating the average of the egg counts from the triplicate urine filtration. Infection intensity was classified following WHO cutoffs [20].

Chi-squared analyses were performed to determine the associations between different markers of morbidity by sex, age, intensity of infection or markers of UT infections.

## Results

### Study flow

**Fig 1:**
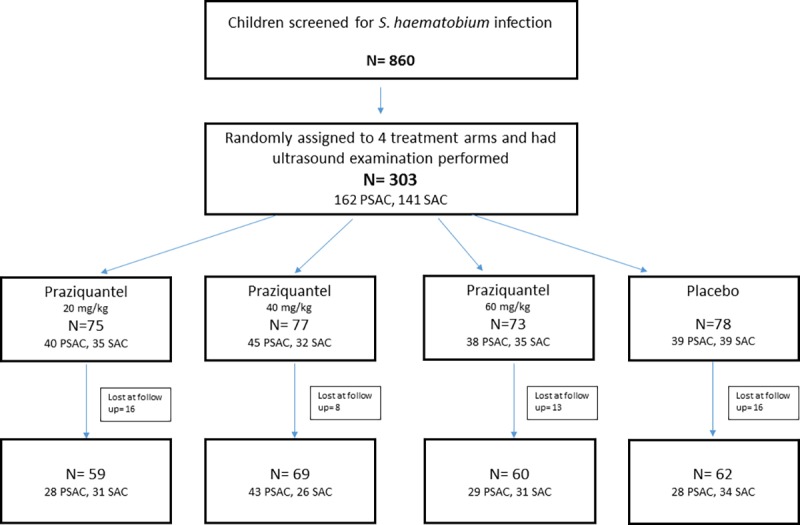
Flowchart of the study conducted in the Adzopè region of Côte d’Ivoire between November 2015 and May 2016.

In November 2015 303 of the 348 children enrolled in the randomized controlled trial underwent an evaluation of the UT with ultrasound (Fig 1). Demographic, clinical and parasitological baseline data are presented in Table 1. Briefly, 162 of the 303 children were PSAC with a mean age of 3.8 (2–5) years. 46% of the preschoolers were male. 141 participants were SAC. Their average age was 8.9 (6–15) years and 44% were male. Six months after treatment (May 2016) 250 children (128 PSAC and 122 SAC) had an ultrasonography done for evaluation of UT lesions.

**Table 1:** Baseline characteristics of children infected with *S*. *haematobium* stratified by treatment group. The study was conducted in the Adzopè region of Côte d’Ivoire in November, 2015.

		Preschool-aged children					School-aged children					Total
Baseline		Placebo	20 mg/kg	40 mg/kg	60 mg/kg	Total	Placebo	20 mg/kg	40 mg/kg	60 mg/kg	Total	
		N = 39	N = 40	N = 45	N = 38	N = 162	N = 39	N = 35	N = 32	N = 35	N = 141	N = 303
Demography	N Male (%)	16 (41.0)	18 (45.0)	25 (55.6)	15 (39.5)	74 (45.7)	16 (41.0)	18 (51.4)	14 (43.8)	13 (37.1)	61 (43.3)	135 (44.6)
	Age (SE)	3.8 (0.2)	3.7 (0.2)	4 (0.2)	4.0 (0.2)	3.8 (0.1)	8.4 (0.4)	8.9 (0.4)	9.3 (0.4)	9.2 (0.5)	8.9 (0.2)	6.2 (0.2)*
	Weight (kg) (SE)	14.9 (0.6)	15.1 (0.4)	15.3 (0.4)	15.4 (0.3)	15.2 (0.2)	23.2 (0.8)	24.1 (0.8)	25.1 (1.2)	25.7 (1.5)	24.4 (0.5)	19.5 (0.4)
	Height (cm) (SE)	97.9 (2.2)	99.0 (1.6)	101.2 (1.7)	99.6 (1.5)	99.5 (0.9)	125.2 (1.7)	127.3 (1.4)	126.9 (2.1)	127.3 (2.3)	126.6 (0.9)	112.2 (1.0)
*S*. *haematobium* infection	Light infection intensity N (%)	34 (87.2)	36 (90.0)	38 (84.4)	34 (87.2)	142 (87.7)	26 (66.7)	23 (65.7)	23 (71.9)	26 (74.3)	98 (69.5)	239 (78.8)
	High infection intensity N (%)	5 (12.8)	2 (5.0)	6 (13.3)	4 (10.5)	17 (10.5)	13 (33.3)	12 (34.3)	9 (28.1)	9 (25.7)	43 (30.3)	60 (19.8)
	EPG AM (CI 95%)	21.8 (8–35.6)	11.9 (6.9–16.9)	20.8 (10.6–30.9)	19.4 (12.5–26.2)	18.5 (13.8–23.2)	91.9 (21.2–162.6)	114.8 (0–252.4)	10.4 (22–58.8)	37.7 (20.7–54.8)	71.6 (33.7–109.5)	43.1 (25.2–61)
	EPG GM (CI 95%)	7.6 (4.7–11.9)	5.9 (3.7–8.9)	8.1 (5.2–12.5)	10.1 (6.4–15.5)	7.8 (6.2–9.6)	32.7 (21.4–49.7)	30.1 (18.7–48.1)	22.9 (15.5–33.5)	19.5 (12.8–29.3)	25.8 (21–31.7)	13.7 (11.7–16.1)
Co-infections	Soil-transmitted helminths	0 (0.0)	0 (0.0)	0 (0.0)	0 (0.0)	0 (0.0)	0 (0.0)	0 (0.0)	0 (0.0)	0 (0.0)	0 (0.0)	0 (0.0)
	*S*. *mansoni*	2 (6.5)	7 (20.0)	9 (23.1)	6 (18.2)	24 (18.5)	0 (0.0)	3 (9.4)	0 (0.0)	1 (3.1)	4 (2.8)	28 (9.2)
	*P*. *falciparum*	15 (38.5)	16 (40.0)	20 (44.4)	13 (34.2)	64 (39.5)	16 (41.0)	14 (41.2)	17 (53.1)	12 (35.3)	59 (41.8)	123 (40.6)
Clinical findings	Hemoglobin (g/dl) (SE)	10.7 (0.2)	10.8 (0.2)	10.5 (0.2)	10.9 (0.2)	10.7 (0.1)	11.3 (0.2)	11.1 (0.2)	11.4 (0.2)	11.7 (0.2)	11.4 (0.1)	11 (0.1)
	Hematuria N (%)	23 (65.7)	21 (56.7)	24 (53.3)	25 (73.5)	93 (61.6)	34 (87.2)	26 (78.8)	22 (73.3)	25 (75.8)	107 (75.9)	200 (66.0)
	Proteinuria N (%)	11 (31.4)	11 (29.7)	18 (40)	16 (47.1)	56 (37.1)	29 (74.4)	25 (75.8)	26 (86.7)	26 (78.8)	106 (75.2)	162 (53.5)
	Leukocyturia N (%)	13 (37.1)	19 (51.4)	18 (40)	23 (67.7)	73 (48.3)	29 (74.4)	25 (75.8)	20 (66.7)	27 (81.8)	101 (71.6)	174 (57.4)
	Nitrituria N (%)	10 (28.6)	3 (8.1)	5 (11.1)	5 (14.7)	23 (15.2)	1 (2.6)	1 (3.0)	0 (0.0)	2 (6.1)	4 (2.8)	27 (8.9)
	Urinary tract morbidity N (%)	19 (48.7)	12 (30.0)	20 (44.4)	19 (50.0)	70 (43.2)	28 (71.8)	23 (65.7)	19 (59.4)	23 (65.7)	94 (66.7)	164 (54.1)*
	Urinary tract morbidity light N (%)	18 (46.2)	13 (30.0)	18 (40)	17 (44.7)	65 (40.1)	21 (53.9)	16 (45.7)	14 (43.8)	15 (42.9)	66 (46.8)	131 (43.2)
	Urinary tract morbidity severe N (%)	1 (2.6)	0 (0.0)	2 (4.4)	2 (5.3)	5 (3.1)	7 (18.0)	7 (20.0)	5 (15.6)	8 (22.9)	27 (19.1)	32 (10.6)
	Polyps/mass morbidity N (%)	1 (2.6)	1 (2.5)	1 (2.3)	0 (0.0)	3 (1.8)	1 (2.6)	1 (2.9)	2 (6.3)	2 (5.7)	6 (4.2)	9 (3.0)
	Kidney morbidity N (%)	4 (10.3)	2 (5.0)	4 (8.9)	1 (2.6)	11 (6.8)	2 (5.1)	4 (11.4)	1 (3.1)	2 (5.7)	9 (6.4)	20 (6.6)

* Significant correlation (p<0.05) between urinary tract morbidity at baseline and age. SE: standard error

### Clinical symptoms and parasitology

#### Baseline characteristics

88% (142/162) of PSAC had a light *S*. *haematobium* infection, with a geometric mean of 7.8 eggs per 10 ml urine. 40% had a co-infection with *P*. *falciparum* and 18.5% with *S*. *mansoni*, no co-infection with soil-transmitted helminths was found.

Urine analysis revealed that 62% of PSAC had microhematuria, 48% had leukocyturia, 37% had proteinuria and 15% had nitrites in urine (Table 1). The most common clinical symptoms among the PSAC at baseline were cough (32%) and headache (22%). Physical examination revealed that 36% had palpable splenomegaly and 33% hepatomegaly.

70% (98/141) of SAC had a light *S*. *haematobium* infection, with a geometric mean of 25.8 eggs per 10 ml urine. 42% had a co-infection with *P*. *falciparum* and 3% were co-infected with *S*. *mansoni*, no co-infection with soil-transmitted helminths was found. Urine examination revealed that 76% of SAC were positive for microhematuria, 72% revealed leukocyturia, 75% showed proteinuria and 3% had nitrites in their urines. When asked about presence of symptoms, 23% of SAC reported cough, 18% had fever and 16% documented headache. 28% had palpable splenomegaly and 34% hepatomegaly (Table 1).

#### Follow up at 6 months after treatment

Six months after treatment 45% of PSAC (ranging from 35% in the 40 mg/kg treatment arm to 64% in the placebo group) were positive for *S*. *haematobium* based on urine filtration with a geometric mean of 1.5 eggs/10 ml urine. 14.3% and 7% of PSAC who received placebo and 20 mg/kg praziquantel, respectively were characterized by high intensity of infection in contrast to children treated with 40 and 60 mg/kg praziquantel. Analysis of the urine revealed that 33% had microhematuria, 53% had leukocyturia, 6% had proteinuria and 48% had nitrites in their urines. In the placebo group hematuria was more prevalent (41%) than in the 60 mg/kg praziquantel treatment arm (29%). For the other chemical parameters the difference among the treatment arms was less pronounced (Table 2).

**Table 2:** Follow up characteristics of children infected with *S*. *haematobium* assessed 6 months after treatment and stratified by treatment group.

		Pre-school aged children						School-aged children					Total
Follow up		Placebo	20 mg/kg		40 mg/kg	60 mg/kg	Total	Placebo	20 mg/kg	40 mg/kg	60 mg/kg	Total	
		N = 28	N = 28	N = 43		N = 29	N = 128	N = 34	N = 31	N = 26	N = 31	N = 122	N = 250
*S*. *haematobium* infection	Positive N (%)	18 (64.3)	13 (46.4)	15 (34.9)		12 (41.4)	58 (45.3)	28 (82.4)	15 (48.4)	11 (42.3)	17 (54.8)	71 (58.2)	129 (51.6) ^3^
	Light infection intensity N (%)	14 (50)	11 (39.3)	15 (34.9)		12 (41.4)	52 (40.6)	20 (58.8)	15 (48.4)	11 (42.3)	16 (51.6)	62 (50.8)	114 (45.6) ^1^^,^^3^
	High infection intensity N (%)	4 (14.3)	2 (7.1)	0 (0.0)		0 (0.0)	6 (4.7)	8 (23.5)	0 (0.0)	0 (0.0)	1 (3.2)	9 (7.4)	15 (6) ^1^^,^ ^3^
	EPG AM (CI 95%)	17.3 (6.5–28.1)	5.7 (1.3–10.2)	2.7 (0.5–4.8)		2.6 (0.5–4.7)	6.5 (3.7–9.2)	42.2 (21.1–63.2)	3.3 (0.8–5.7)	2.8 (0.2–5.4)	5.2 (0.1–10.4)	14.4 (7.8–20.9)	10.4 (6.8–13.9)
	EPG GM (CI 95%)	5.0 (2.2–10.5)	1.6 (0.5–3.4)	0.7 (0.2–1.3)		0.9 (0.3–1.7)	1.5 (1–2.2)	12.2 (6.1–23.3)	1.2 (0.5–2.1)	0.9 (80.3–1.9)	1.3 (0.5–2.4)	2.5 (1.7–3.6)	2 (1.4–2.5)
Clinical findings	Hematuria N (%)	9 (40.9)	8 (36.4)	11 (30.6)		7 (29.2)	35 (33.4)	20 (64.5)	7 (28.0)	9 (39.1)	10 (34.5)	46 (42.2)	81 (32.4)^2^^,^^3^^,^^4^
	Proteinuria N (%)	1 (4.6)	2 (9.1)	2 (5.6)		1 (4.2)	6 (5.8)	2 (6.5)	3 (12.0)	3 (12.0)	2 (6.9)	10 (8.1)	16 (6.4)
	Leukocyturia N (%)	13 (59.1)	14 (63.6)	17 (47.2)		11 (47.8)	55 (53.4)	17 (54.8)	10 (40.0)	8 (32.0)	9 (32.1)	44 (40.0)	99 (39.6)^4^
	Nitrituria N (%)	10 (45.5)	10 (45.5)	17 (47.2)		13 (54.2)	50 (48.1)	10 (32.3)	5 (20.0)	6 (24.0)	5 (17.9)	26 (23.6)	76 (30.5)
	Urinary tract morbidity N (%)	16 (57.1)	12 (42.9)	23 (53.5)		13 (44.8)	64 (50)	26 (76.5)	16 (51.6)	15 (57.7)	14 (45.2)	71 (58.2)	135 (54.0)^4^ §
	Urinary tract morbidity light N (%)	12 (42.9)	11 (39.3)	21 (48.8)		13 (44.8)	57 (44.5)	19 (55.9)	15 (48.4)	12 (46.2)	12 (38.7)	58 (47.5)	115 (46.0)
	Urinary tract morbidity severe N (%)	4 (14.3)	1 (3.6)	2 (4.7)		0 (0.0)	7 (5.5)	7 (20.6)	1 (3.2)	3 (11.5)	2 (6.5)	13 (10.6)	20 (8.0)
	Polyps/mass morbidity N (%)	1 (3.6)	2 (7.1)	4 (9.3)		0 (0.0)	7 (5.5)	6 (17.6)	2 (6.4)	0 (0.0)	0 (0.0)	8 (6.5)	15 (6.0)
	Kidney morbidity N (%)	1 (3.6)	2 (7.1)	1 (4.7)		1 (3.5)	6 (4.7)	4 (11.8)	1 (3.2)	1 (3.9)	1 (3.2)	7 (5.7)	13 (5.2)

^1^ Significant correlation (p<0.05) between *S*. *haematobium* intensity of infection, hematuria, leucocyturia at baseline and *S*. *haematobium* intensity of infection at follow up

^2 ^Statistical significant correlation (p<0.05) between *S*. *haematobium* intensity of infection at baseline and hematuria at follow up

^3^ Statistical significant correlation (p<0.05) between *S*. *haematobium* intensity of infection and hematuria at follow up

^4^Statistical significant correlation (p<0.05) between UT morbidity at follow up and *S*. *haematobium* intensity of infection, hematuria and leucocyturia at follow up

§ Statistical significant correlation (p<0.005) between UT morbidity and different treatment dosage

During physical examination the most frequently observed symptoms were cough (38%) and fever (19%). 13% of PSAC had palpable splenomegaly and 3% had hepatomegaly.

58% (71/122) of SAC were positive for *S*. *haematobium* based on urine filtration (geometric mean of 2.5 eggs/10 ml urine), ranging from 42% in the 40 mg/kg treatment arm to 82% in the placebo group. Infections were mostly light, with high intensity of infection mainly observed in the placebo group (24%). Analysis of the urine revealed that 42% of children had microhematuria, of which 65% were treated with placebo compared to 28% of children treated with 20 mg/kg praziquantel. 40% of children had leukocyturia, 8% had proteinuria and 24% had nitrites in their urines (Table 2).

At physical examination, the most common symptoms reported were cough (38%) and headache (22%). 9% of SAC had palpable splenomegaly, while none had hepatomegaly.

#### Ultrasound analysis

**Fig 2:**
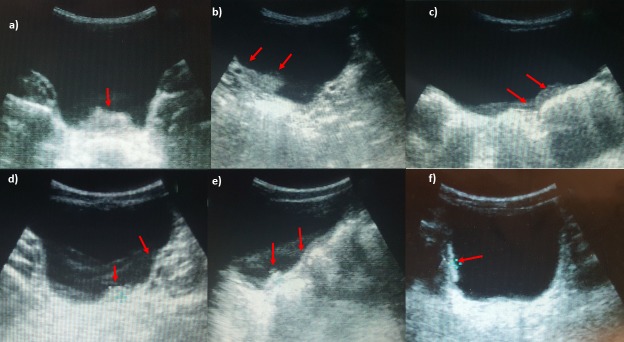
Ultrasonography images of urinary bladder of PSAC infected with *S*. *haematobium*. a) focal thickening of the bladder wall, longitudinal plane shows mass-like lesion (arrows); b) transverse plane image shows diffuse thickening of the bladder wall more evident in the right posterior wall; c) transverse plane image shows a focal heterogeneous echo of the bladder wall in absence of true thickening or mass-like lesions in the lumen; d) longitudinal plane shows a marked and diffuse thickening of the bladder left wall with a mass like lesion (arrow); e) image of diffuse and marked thickening of the bladder wall with pseudo-polyp lesion; f) focal thickening of the wall evident on the right wall and diffuse heterogeneity of bladder echo.

At baseline 43% (70/162) of PSAC had UT morbidity (Fig 2). The vast majority had light/ moderate bladder morbidity (40%), such as focal wall thickening or bladder heterogeneously echoic (Fig 2C and 2F), 2% (3/162) had polyps or masses on their bladder (Fig 2A, 2B, 2D and 2E) and 7% (11/162) had hydronephrosis (Table 1).

**Fig 3:**
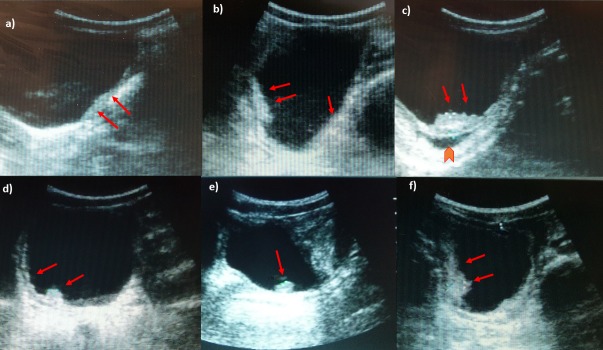
Ultrasonography images of urinary bladder of SAC infected with *S. haematobium*. a) Thickening of the bladder wall, transverse plane shows thickening of the left lateral wall (arrows); b) Diffuse thickening of the bladder wall more evident in the right posterior wall, echogenic snow in the lumen; c) In oblique longitudinal plane ultrasound image shows a mass-like lesion in the mucosa layer of the bladder. Block arrow indicates the dilation of the ureter; d) longitudinal plane shows a marked and diffuse thickening of the bladder wall with a mass-like lesion (arrow); e) mass-like lesion in the absence of a marked and diffuse thickening of the bladder wall; f) multifocal thickening of the wall, particularly evident on the right and posterior wall.

67% (94/14) of SAC presented UT pathology (Fig 3). The majority had light/moderate pathology (47%), such as heterogenous wall or focal thickening of the bladder (Fig 3A, 3B and 3F), 4% (6/141) presented polyps or masses on the bladder wall (Fig 3C, 3D, 3E and 3F) or dilated ureter (Fig 3C) and 6% (9/141) had pyelectasis.

30% of PSAC (48/162) and 1.4% of SAC (2/141) did not reach an adequate fill of the bladder to be analyzed with sonography.

At the six month follow up, 50% (64/128) of PSAC presented morbidity at bladder level, most of it (45%) was mild (thick/ semi-sonolucent mucosa), 6% (7/128) had polyps on the bladder wall and 5% (6/128) had pyelectasis. 58% (71/122) of SAC presented morbidity at bladder level, most of it (48%) was mild (thick/ semi-sonolucent mucosa), 7% (8/122) had polyps on the bladder wall and 6% (7/122) had kidneys dilatation.

Statistical analyses revealed significant correlations between UT morbidity and urine examination (leucocyturia and hematuria) and with intensity of infection both at baseline and at follow up (Table 2).

#### Impact of praziquantel dose on morbidity

19% (24/128) of PSAC and 22% (27/122) of SAC did not have any pathology at sonographic UT examination at baseline. A significant correlation (p<0.05) was observed between praziquantel treatment and reversal of *S*. *haematobium* induced morbidity in all children. In more detail and stratified according to treatment arm, among PSAC who did not experience any resolution of lesion at follow up 10% (2/21) had received 20 mg/kg, 43% (9/21) received 40 mg/kg, 23.8% (5/21) received 60 mg/kg praziquantel and 24% (5/21) placebo. 20 PSAC had worse lesions at follow up. Among these 20% (4/20) received 20 mg/kg, 25% (5/20) 40 mg/kg, 10% (2/20) 60 mg/kg of praziquantel and 45% (9/20) received placebo.

On the other hand, 23 PSAC had an improvement at sonographic follow-up, of which 21.7% (5/23) received 20 mg/kg of praziquantel, 30.4% (7/23) 40 mg/kg, 34.8% (8/23) 60 mg/kg and 13% (3/23) placebo.

Among SAC who did not experience any improvement of the lesion at 6 months follow up, 22.2% (6/27) received 20 mg/kg of praziquantel, 11.1% (3/27) received 40 mg/kg praziquantel, 25.9% (7/27) received 60 mg/kg praziquantel and 40.7% (11/27) received placebo. 45 SAC had an improvement of the UT at follow-up: 33.3% (15/45) received 20 mg/kg of praziquantel, 24.4% (11/45) 40 mg/kg, 26.7% (12/45) 60 mg/kg and 13.3% (6/45) placebo.

22 SAC had aggravated lesions at follow up: among these 13.6% (3/22) received 20 mg/kg, 18.2% (4/22) 40 mg/kg, 4.5% (1/22) 60 mg/kg of praziquantel and 63.6% (14/22) received placebo.

#### Residual urine measurements

Residual volume of urines after bladder voiding was measured both at baseline and at follow up. At baseline 82% (133/162) of PSAC had a low post-void residual (<20%) in the bladder, which means that there is no or very light impairment of the detrusor muscle. Only 2 children had a post-void residual higher than 50%. 12% (20/162) of PSAC had an intermediate (50–80%) value.

At the six months follow up, 91% (117/128) of PSAC had a post -void residual lower than 20%. 2 children still had high residual volume in bladder (>50% of the initial).

86% (122/141) of SAC had a good control performance of the detrusor muscle and only 1 child had a urine residual after bladder void higher than 50%. Almost 10% (14/141) of SAC had a post -void residual between 50 and 20%. At the six months follow up 96% (117/122) of SAC had a normal voiding of the bladder and only in one we registered a post-void volume higher than 50% of the initial one. We did not find any statistical difference between the treatment and improvement of bladder voiding.

## Discussion

To our knowledge this is the first study that analyses urinary tract morbidity in school-aged and preschool-aged children affected by *S*. *haematobium* at baseline and six months after treatment with different praziquantel dosages and placebo. In settings where control of morbidity is the main goal of public health interventions, the most widely used criteria to determine it is the measurement of egg counts and urine analyses for hematuria and proteinuria, as indirect signs of UT impairment [3,12]. However, obviously a more accurate and specific evaluation of the organ pathology should be the way to follow [12,21–22]. Ultrasound examination allows to assess the damage of bladder wall and genito-urinary tract, which in combination with parasitological results and urine analyses are good indicators of consequences of chronic infection [4,12,13]. Ultrasonography has been applied since the ‘70s [21] for schistosomiasis to detect and describe the morphology of lesions. The need to implement diagnostic and monitoring with ultrasound is widely shared [4,10,21], but so far its use is still limited [21].

Since in schistosomiasis UT morbidity often occurs asymptomatic until an advanced grade of pathology [23,24], ultrasound offers the great advantage to spot early complications and progression of pathology in a non-invasive and easy to perform manner.

Our study confirms that early complications and bladder consequences of a *S*. *haematobium* infection are frequent also in preschool-aged children [5] (Fig 2). We recorded both direct and indirect signs of infection that give a full and detailed picture of UT status in infected children of different ages. In more detail, in our study most children (79%) had low intensity infection but nonetheless of these 54% of children (43% of PSAC and 67% of SAC) presented UT morbidities. As Hatz and colleagues pointed out [4], lesions of the bladder are observed also in absence of excretion of eggs, as these might be stuck and trapped in the wall resulting in an inflammatory reaction, that does not allow their release. Also for other helminthic infections, it has been demonstrated that morbidity (such as anemia, stunting) is mostly triggered by chronicity of infection rather than by its intensity [25,26].

According to our findings, children are not affected by severe morbidity, in fact, the greater part of hydronephrosis resolved immediately after urination. We also did not observe a frequent presence of pseudopolyps or masses in the bladder (6%). Our data are in line with findings by Koukounari and Njaanake [10,27], but in contrast to Elmadani, who described that more than 40% of children had masses in the bladder lumen and 30% had hydronephrosis after urination [13]. The prevalence of UT morbidity is indeed very different from one study to the other. For example, Heutier and colleagues registered a 70% prevalence of bladder lesions in an African village endemic for *S*. *haematobium* in children [28], whereas Ekwunife and Koukounari reported a lower rate of UT morbidity in infected children (38% and 6% respectively) similar to what we have found [10,12].

As already reported and underlined in several trials on *Schistosoma* morbidity [4,18,29–31], praziquantel treatment is crucial in decreasing morbidity with regard to healing lesions and pathology linked to the infection, especially at early stages of the disease. In the present study we went a step further and studied the effect of different praziquantel doses and placebo on UT morbidity. Strikingly, while in the placebo group almost 40% of children had progression of UT pathology over the 6 months course, this rate decreased with increasing dosages being only 5% in the children treated with 60 mg/kg praziquantel. In addition, all dosages of the drug were correlated with an improvement of the clinical picture. Overall, more than 90% of treated children experienced improvement of lesions, whereas in the placebo group this rate was only 10%.

In our study 74% of children had no residual urine after bladder void and 12% had a residual volume greater than 50%. We did not perform an uroflowrimetry to confirm pathological voiding, but children were asked about symptoms linked to urination discomfort and reported urge of voiding even if the bladder was almost empty and a feeling of incomplete voiding was present. Voiding impairment is difficult to assess and confirm, especially in children and in conditions of stress such as ultrasound performance and clinical examination. Nonetheless, we observed an improvement at follow up both in bladder filling and discomfort in urination, though this was not properly validated. Akpata stated that the above mentioned symptoms are better indicators of schistosomiasis than residual volume calculation [21]. Stiffness of detrusor muscle, polyps and hydronephrosis are signs of severe stage of the pathology, which fortunately was rare in our study cohort. This suggests that the annual drug administration that takes place in the area is a good strategy to fight morbidity and decrease UT impairment [4,27].

Urinary tract infections (UTI) are common in childhood, accounting for 6% of infection in this age range [32]. In our study leucocyturia was often documented (57%), especially among SAC (72% *versus* 48% in PSAC), whereas nitrituria was more frequent among PSAC (15 vs 3%) [33,34]. According to our data, lower urinary tract morbidity was correlated with a general worsening of the UT, revealing a higher rate of nitrates and proteins and blood cells in urines, which is an evident sign of mucosa damage. As in previous trials, we also found hematuria and proteinuria to be good indicators of UT pathology (66% and 56% respectively) [10,15]. The prevalence of UTI in our study was higher than earlier findings for same age group [32], but this is not surprising given the fact that almost half of the children had chronic infections with *S*. *haematobium*. Chronic infections with *S*. *haematobium* are the main cause of mucosa damage and hence more likely to develop subsequent bacterial infections [35]. After treatment we observed an improvement of microhematuria in treated children compared to placebo treated children in both age groups.

On the other hand, clinical symptoms documented, such as fever and cough, were not found to be related to an *S*. *haematobium* infection, but are likely triggered by other diseases. For instance, at physical examination splenomegaly and hepatomegaly was observed in 30% of children, which might be correlated to *S*. *mansoni* or to other co-infections (e.g. malaria, leishmaniasis).

In conclusion, we have demonstrated that extending treatment (40 or 60 mg/kg praziquantel) from school-aged to preschool-aged children is crucial, in order to prevent morbidity to a *S*. *haematobium* infection. We have shown that already a high percentage of PSAC present bladder inflammation and mucosa thickening due to *S*. *haematobium* infection, which could be decreased by including this age group in treatment programs. We observed a high re-infection rate with *S*. *haematobium*, therefore preventive chemotherapy must be conducted at least once a year in PSAC and SAC in order to decrease morbidity.
